# Long-Term Complications of Chronic Traumatic Paraplegia: An Experience from Pakistan

**DOI:** 10.7759/cureus.116

**Published:** 2013-05-15

**Authors:** Ahmed Bakhsh

**Affiliations:** 1 Neurosurgrey, Saad Specialist Hospital, Riyadh, SAU

**Keywords:** quality of life, stem cells, probiotics, spinal cord injury, paraplegia, pressure sores, urinary tract infection, rehabilitation, complications, prognosis

## Abstract

Objective: This study was conducted to ascertain the long-term complications of chronic traumatic paraplegia and the quality of life of paraplegic patients.

Study Design: A retrospective descriptive study.

Place and duration of study: Conducted at Fauji Foundation Hospital, Rawalpindi, Pakistan.

Materials and Methods: Twenty-six admitted male patients suffering from traumatic paraplegia were physically and neurologically examined, and the available laboratory and radiological investigations were done. The medical records of all patients were thoroughly reviewed.

Results: Falls were found to be the most common cause of the paraplegia (57.7%). Neurological recovery did not occur in any patient, even after three decades. All patients had developed complications of urinary tract infections, such as chronic renal failure, renal/ bladder stones, and epidydimo-orchitis. Urinalysis showed asymptomatic bacteriuria in all patients. Urine culture showed Pseudomonas Aeruginosa in 65.3% and E. Coli in 42.3% of samples. Multiple uropathogens were present in 77.9% of cases. Persistent and recurrent bed sores were present in 46.1% patients. Many patients had episodic, burning leg pain, spasticity of legs (76.9%), and contractures of knee joints. All patients were irritable, depressed, and had suicidal ideas.

Conclusions: This study showed that traumatic paraplegia is a permanent disability. It is associated with high morbidity rate due to scores of complications, particularly recurrent urinary tract infections and pressure sores. Prevention, early detection, and timely intervention of potential complications are of the utmost importance.

## Introduction

Spinal cord injury is one of the most devastating injuries known to the humanity. The annual worldwide incidence of acute spinal cord injuries is approximately 15-40 per million people [1-2]. Spinal cord injuries cause immense economic, social, and psychological burden both on the families as well as on the society. Its annual cost in the USA is 9.7 billion US dollars per year [3-4].

The available therapies for spinal cord injuries are either limited or ineffective. So far, no permanent cure has been discovered. Study for Surgical Treatment of Acute Spinal Cord Injury (STASCIS) showed only modest benefit of early surgical decompression of spinal cord [5]. Several neuroprotective agents like Methylprednisolone, Tirilizad, Thyrotrophic-releasing hormone, Naloxone, and Nimodipine have been tried in large prospective clinical trials, but without any significant benefit. Trials are still under way for new neuroprotective drugs, such as Riluzole (Sodium-Glutamate Antagonist), Minocycline, anti-Nogo, and anti-Rho substances [6].

Stem cell therapy has also been found potentially effective only for acute spinal cord injuries. Chronically injured spinal cords are not hospitable for the transplanted cells due to the complex pathophysiology, glial scarring, and syrinx formation. Therefore, the future of the patients with chronic spinal injuries is still bleak [7].

At all stages, spinal cord injuries are fraught with numerous medical complications. Some complications like pyelonephritis, ureteric colic, acute abdomen, hip fracture, and deep veins thrombosis may present without any typical signs and symptoms due to sensory loss and are therefore likely to be overlooked easily [4].  Although types of complications are the same all over the globe, but the rate of complications is much higher in developing countries [8].

This study will highlight the severity of the problem and the pathetic quality of life of traumatic paraplegic patients. It will also explain that only residential care and physiotherapy are not enough for paraplegic patients, rather a more comprehensive and holistic clinical approach is desirable [9].

Abbreviations

UTI (urinary tract infection), DVT (deep vein thrombosis), CT/MRI (computerized tomography/magnetic resonance imaging)

## Materials and methods

This study was conducted at Fauji Foundation Hospital, Paraplegic Centre, Rawalpindi, Pakistan. The Fauji Foundation had established this centre primarily for nursing care of male traumatic paraplegic patients. The centre had been providing residence, medical care, rehabilitation, and vocational facilities free of cost. After four decades, the center was closed, with an aim to integrate paraplegic patients into the society.

At the time of discharge, author personally examined all patients with particular emphasis to their neurological status. Review of medical records showed that all patients had sustained spinal cord injuries resulting in paraplegia. All patients had either thoracic or thoracolumbar spinal injuries. No patient had either cervical or lumbar injury. These patients were transferred from other hospitals after their initial resuscitation, stabilization, and conservative management. The time of injury to the time of transfer to this center was not available.

Preliminary investigations, such as complete blood count, liver/renal function tests, urinalysis and culture, chest x-ray, whole spine plain radiography, and retroperitoneal ultrasound, were completed for all patients. Computerized tomography, magnetic resonance imaging of thoracolumbar spine, and bone densitometry were not done in any patient due to financial constraints and the unavailability of some facilities at the centre.

## Results

In total, 26 male paraplegic patients were studied. The majority of the patients (57.69%) were in their fourth and fifth decade.

**Figure 1:**
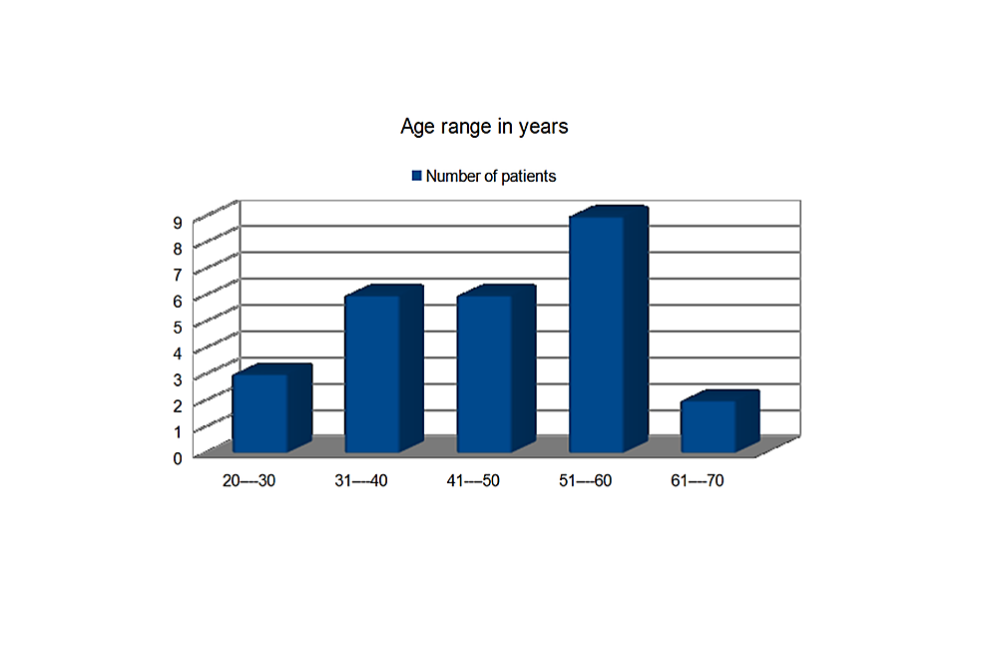
Age Range in Years The common causes of paraplegia were falls (65.38%), road traffic accidents (23.07%) and firearm injuries (11.53%). Falls from hills, trees, and electric poles were some common reasons in this society.

**Figure 2:**
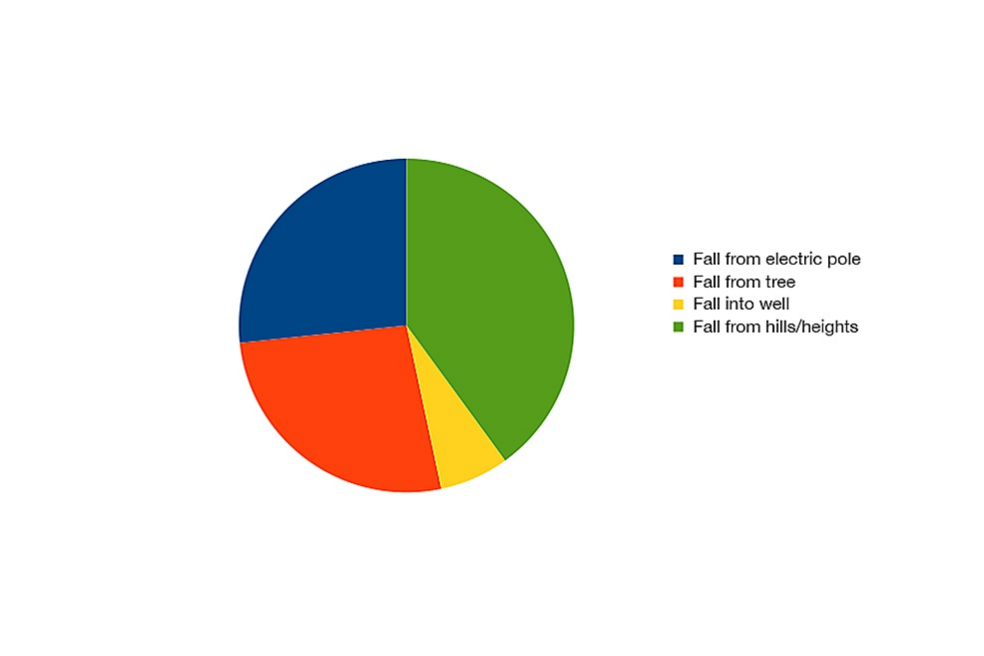
Nature of Falls

The common causes of paraplegia were falls (65.38%), road traffic accidents (23.07%) and firearm injuries (11.53%). Falls from hills, trees, and electric poles were common reasons in this society (Figure 2).

The duration of traumatic paraplegia was more than two decades in 76.92% cases. Local clinical examination of the whole spine did not reveal any visible bony deformity in 69.23% of the cases. In others, gibbous and kyphoscoliosis deformities were quite obvious.

Spastic atrophic legs (power 0/5) were seen in 76.92% and flaccid atrophic legs (power 0/5) in 23.07% patients. More than two-thirds of patients (76.92%) had sensory level up to the umbilicus and the rest had sensory level either below or slightly above the umbilicus. No patient was suffering from chronic cauda equina syndrome. All patients were doubly incontinent.

Few patients (11.53%) had never developed any pressure sores throughout their illness. Pressure sores developed initially in 42.30% cases, but later healed. Nearly one-half (46.15%) of the patients had persistent and recurrent grade 1V pressure sores. A few patients also underwent grafting of pressure sores. Osteomyelitis of hip joints secondary to the bed sores developed in 7.69% patients.

All patients were on intermittent self-catheterization. Every patient had been experiencing one to two episodes of recurrent urinary tract infection per month. Urinary tract infection was considered only if patient had developed symptoms of infection, like fever, and laboratory evidence of urinary tract infection, like high erythrocyte sedimentation rate, leucocytosis, pyuria, and positive urine culture. Urinalysis and culture showed bacteriuria in all but multiple pathogens in 76.92% patients. Many patients had developed complications of repeated urinary tract infections.

**Table 1:** Urinary Pathogens

Name of Bacteria	Number of Patients
Pseudomonas aeruginosa	17
E.coli	11
Klebsiella pneumonie	08
Staphylococcus aeureus	07
Enterobacteria	05
Streptococcus faecalis	04
Proteus vulgaris/morgagni	07
Streptococcus pyogens	01

**Table 2:** Sequelae of UTI

Hydronephrosis	08 (30.76%)
Dysuria/ pyuria/ haematuria	26 (100%)
Renal calculi	04 (15.38%)
Epididymo-orchitis	03 (11.53%)
Hypertension	04 (15.38%)
Chronic renal failure	03 (11.53%)
Bladder calculi	04 (11.53%)

**Figure 3:**
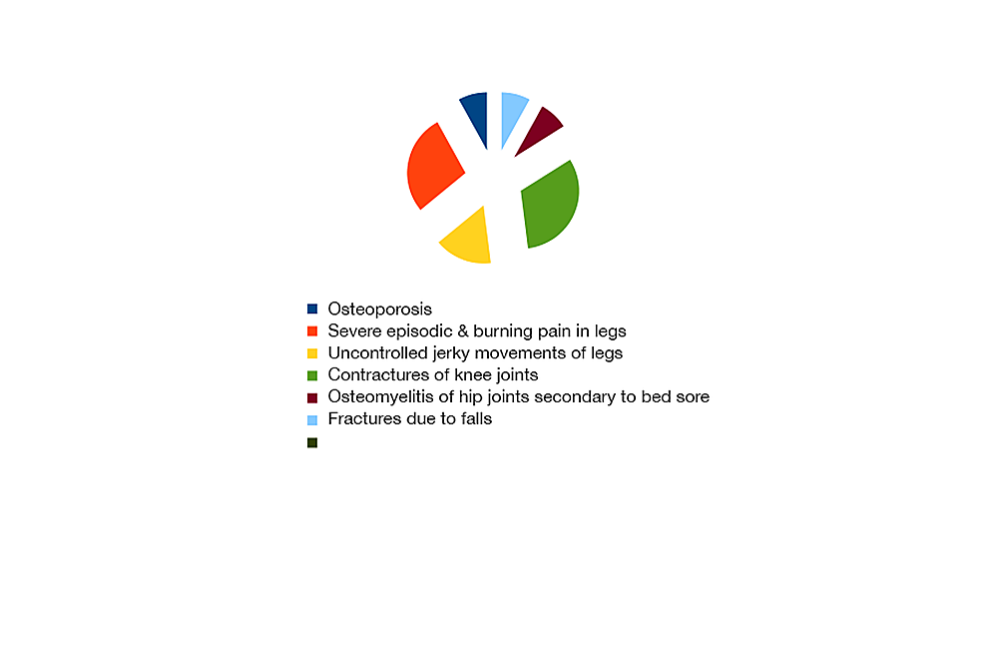
General health of all patients was found very poor. Dental, mouth, skin infections, and various gastrointestinal ailments were some common problems. Many patients needed different kinds of surgeries during their hospital stay as well.

Severe episodic and burning leg pain was recorded in 26.92% and uncontrolled jerky legs movements and contracture of knee joints in 30.76% patients (Figure 3).

General health of all patients was found very poor. Dental, mouth, skin infections, and various gastrointestinal ailments were some common problems. Many patients needed different kinds of surgeries during their hospital stay as well.

**Figure 4:**
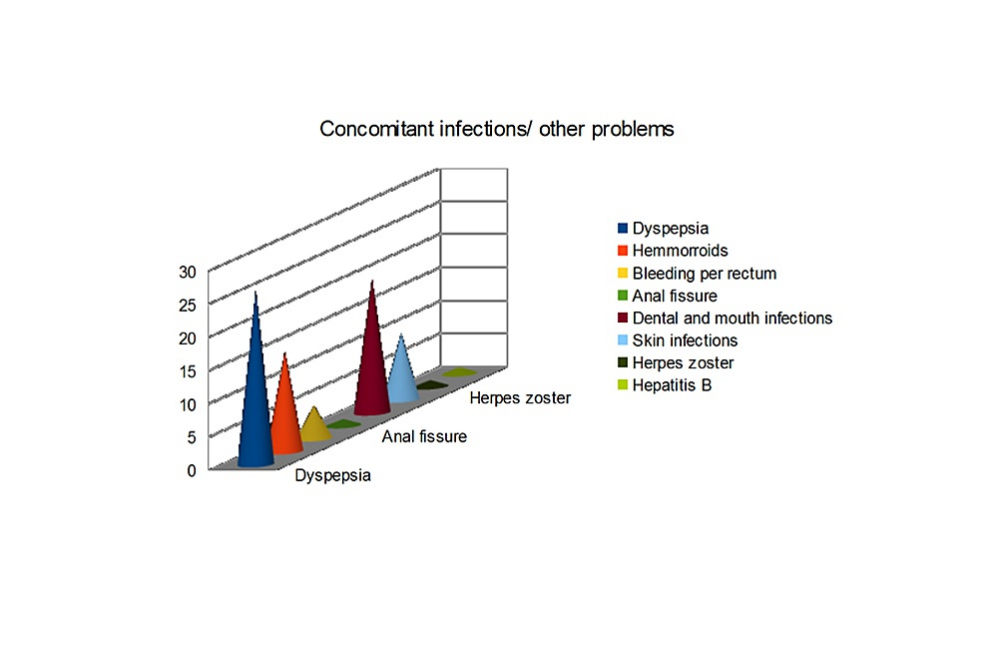
Concomitant Infections / Other Problems

**Figure 5:**
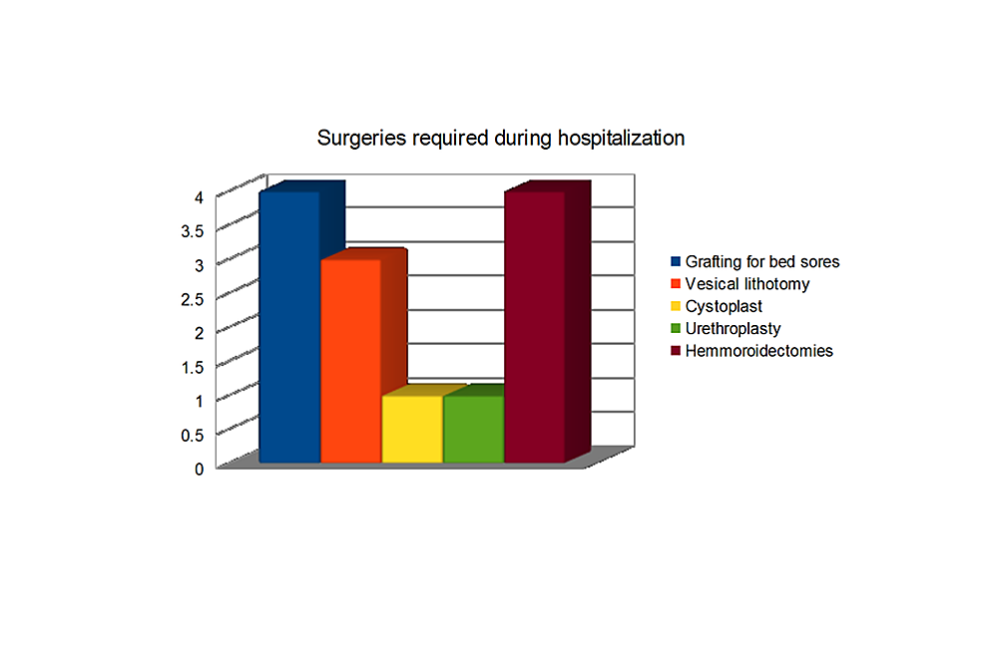
Surgeries Required During Hospitalization

Plain x-ray of thoracolumbar spine, anteroposterior and lateral views were done in all patients. Because of advanced vertebral deformities and osteoporosis of the spine, exact anatomical level of spinal injury could not be determined on the basis of these images.

## Discussion

Across the world, young males are more prone to spinal injuries. This study did not consist of any female patients because the centre was exclusively for male paraplegic patients. Little information is available in the literature regarding the true global incidence of traumatic paraplegia in females. Nonetheless, male preponderance and mean age of our patients (45 years) were quite consistent with the international literature [7].

Falls from height, followed by road traffic accidents, remained the leading cause of paraplegia in this study. In developed countries, road traffic accidents are the predominant cause of traumatic paraplegia, but in developing countries, falls have been reported as the most common cause of spinal cord injury [8].

Urinary tract infections and spinal cord injury always go together. Within eight hours after urinary catheterization, a biofilm is formed around the catheter which keeps the bacteria dormant and alive. Within seven days, 77% patients develop urinary tract infections [10].  According to one study, 100% patients developed urinary tract infections soon after the admission [11]. Before the advent of antibiotics, more than 80% paraplegics used to die due to renal failure. Nowadays, better nursing and medical care, as well as improved diagnostic tools, have significantly reduced the mortality rate [12].

Paraplegics are unable to empty urinary bladder due to detrusor hyperactivity, sphincter hyperactivity, detrusor-sphincter dyssynergia, or flaccid bladders. Frequent use of urinary catheters, raised intravesical pressure, and increased post-void residual urine volume are the main reasons of urinary tract infection [13]. Once infection is established, it is usually persistent and resistant to the antibiotics. Therefore, renal failure and septicemia are perpetual risks for all such patients [14].

At the time of this study, no patient had any overt urinary tract infection, but the history of monthly episodes of recurrent UTI in all patients was indeed alarming. More than quarter patients had already developed hydronephrosis and urolithiasis.

The incidence of asymptomatic bacteriuria is 70% in self-catheterization and 98% in indwelling catheterization. It can give rise to the complications like pyelonephritis, renal/bladder stones, renal failure, and death [1, 14]. In this study, 100% patients were suffering from asymptomatic bacteriuria. Pseudomonas aeruginosa was the commonly isolated organism followed by E. coli. Other studies reported E. coli, Enterococci, and Klebsiella pneumoniae as frequently cultured uropathogens [15]. Bosnian study reported Providencia and Proteus as the frequent isolated organisms [10]. Pseudomonas aeruginosa has been considered colonizers rather than uropathogens, but in one study, it was reported as the most common organism [14].

Controversy still exists whether asymptomatic bacteriuria should be treated or not. Asymptomatic bacteriuria with or without pyuria does not warrant any antibiotic therapy. Antibiotics may be justified for Proteus mirabilis which produces unease and causes renal and bladder stone formation. Weekly oral cyclic antibiotic program (WOCA) has also shown considerable reduction in antibiotics consumption and episodes of urinary tract infection [1].

Prevention of recurrent UTI is certainly challenging. Use of topical/oral antiseptics, acidifying/alkalinizing urinary agents, and various types of urinary catheters, like plastic, silicone, latex, and antibiotic-impregnated, failed to prevent recurrent UTI. Receptor analogues and vaccines are being tested to prevent bacterial adhesions in the uroepithelium. Instillation of mucopolysaccharidases into the bladder similar to naturally acting glycosaminoglycan has also been suggested. Use of Lactobacilli vaginal pessaries has significantly reduced recurrent UTI in females by alternating the local bacterial flora [14]. 

Probiotics are also being explored for the prevention and treatment of recurrent UTI. These are living microorganisms which produce bactericidal or bacteriostatic substances and do exert synergistic effects with antibiotics. Trials of probiotics have given encouraging results for irritable bowel syndrome, Helicobacter pylori infection, and Clostridium difficile positive diarrhea. Probiotics cannot completely eradicate the infection, but may lower the rate of recurrence. E. Coli HU2117-coated urinary catheters and instillation of benign E. coli strain into the bladder have shown significant reduction in recurrent UTI [16].

Surgical and mechanical means are also available to prevent recurrent UTI. Normal low-pressure reservoir function of the bladder is created by resecting the bladder above the trigone and substituted with an ileum and colon graft. Sacral nerve stimulation and pharmacological agents acting on the bladder and sphincters are also other available options [12].

Decubitus ulcers are common complication of the traumatic paraplegia [17]. Pressure sores develop due to an unrelieved pressure over bony prominences causing necrosis of muscles, subcutaneous tissue, dermis, and epidermis. Common risk factors involved are sensory loss, social status, malnutrition, and inadequate nursing care [18]. Two-third pressure sores occur around the pelvis and one-third on the lower limbs. Non-healing wounds reflect the underlying osteomyelitis. The true depth of pressures sores can only be ascertained by CT/MRI imaging [4].

In one study, 39% patients presented with pressure sore at the time of admission [8]. This study showed 46.15% patients had recurrent and persistent bed sores. Gluteal and sacral regions were more commonly involved areas in these cases [18].

Deep veins thrombosis is a frequent complication in acute spinal cord injuries, but its incidence in chronic spinal injuries has been reported quite low [11, 19-20]. Occurrence of deep vein thrombosis in Asian population is usually considered low due to genetic and ethnic variations [21]. In this study, no patient had any sign or symptom of deep vein thrombosis, and no patient was on any prophylactic DVT treatment. Spasticity and contractures might be the reasons for very low risk of DVT in these chronic patients.

Osteoporosis is well-recognized, but overlooked complication of spinal cord injuries. Its exact pathogenesis is unknown, but immobility is the biggest contributing factor. Approximately, one-third of bone mineral density is lost within first three to four months following spinal injury. This increased bone turnover predisposes to osteoporosis, fractures, hypercalcemia, ectopic calcification, and renal calculi [22]. An estimated incidence of fractures after spinal cord injuries is 5-20%. This study also showed advance osteoporosis of the spine in many patients on plain radiography of spine.

Two-thirds of the spinal cord injured patients suffer from chronic pain syndrome [23]. This study showed 26.30% patients were suffering from intractable pain. Neurogenic pain is usually burning, stabbing, or electrical in quality. Although neurogenic pain appears to be associated with neuronal hyper-excitability but exact mechanisms are poorly understood. This pain syndrome is usually resistant to treatment, and it greatly impairs and compromises the quality of life of the patients.

Over 75% patients develop spasticity one year after spinal cord injury. In this study, 76.92% patients had spasticity of legs.  Spasticity occurs frequently in lesions above the conus medullaris. Its pathophysiology is so far unknown. It is believed to result due to disruption of descending inhibitory modulation of the alpha motor neurons, producing hyper-excitability. Baclofen and diazepam are drugs of choice, but severe and resistant spasticity may need surgical treatment [4, 24].

Currently, stem cell therapy is being explored for various developmental, traumatic and degenerative brain disorders. The main aim is to produce new neurons and oligodendrocytes and to induce the axonal regeneration and remyelination [25].  Embryonic stem cells can be obtained without destroying embryonic or fetal life, but immune rejection and potential of tumor growth are still major concerns. Moreover, future recipients might be at risk from previously unknown diseases transmitted through donor cell lines [26].  Currently, genetically reprogrammed, induced Pluripotent Stem Cells (iPSC) have been created by using viral gene transfer into somatic cell of adult humans which are genetically identical to the donor genome. In the future, iPSC are likely to be produced without any viral transduction, and by non-genetic approaches [27]. Fetal neuronal progenitor cells can also differentiate into neurons and oligodendrocytes after transplantation but ethical reasons prevent their large scale use. Mesenchymal stem cell from bone marrow and umbilical cord blood cells are other readily available autologous cells for transplantation. These cells migrate to the site of injury following intravascular and intrathecal administration [6]. Injection of activated autologous macrophages into the injured spinal cord has also proved high degree of safety and efficacy in Food and Drug Administration (FDA)-approved Phase 1 human clinical trial [28].

Researchers are also exploring potential for neural regeneration through induction and manipulation of endogenous stem cells. These cells respond to injury by proliferation and maturation into the astrocytes rather than into oligodendrocytes and neurons. The goal is to alter the cellular fate of these endogenous stem cells for axonal repair. Their proliferation potential can be increased by the addition of stimulating factors like epidermal growth factor, insulin-like growth factor-1, FGF-2, and bone morphogenetic protein [3].

At the site of injury, some potent neurite growth inhibitory proteins like Nogo-A, myelin-associated glycoproteins (MAG), and oligodendrocytes-myelin glycoproteins are produced. These substances pose formidable barrier in recovery of neural tissue. Nogo-A inhibits axonal growth through Rho A and Rac 1 molecules. Use of Rho antagonist (Catherin) has shown considerable neurological improvements after one year of follow-up in human clinical trials. Scars also produce neurite growth inhibitory substances, like chondroitin sulfate proteoglycans. Injection of chondroitinase into lesion sites in the brain or spinal cord has shown increased sprouting of axons [5].

Oscillating field stimulation therapy is another upcoming treatment of acute spinal cord injuries. This technique is based upon observations that electrical signals guide and promote the neuronal growth. In a Phase-1 FDA-approved clinical trial, all patients demonstrated improved sensation and significant motor or sexual functions [29]. 

This is not an ideal study because of certain limitations, like small size of sample, retrospective nature of study, and lack of CT/MRI imaging; nevertheless, it strongly advocates that traumatic paraplegic patients need continuous vigilance and surveillance.

## Conclusions

Recovery of spinal cord injury is still a major challenge for neuroscientists. Hopefully, current intense global research in stem cell therapy will be able to provide safe and effective therapies in the coming years. At present, only prevention, effective nursing and medical care will remain the mainstay in the management of spinal cord injured patients.
